# Phylogenetic and Genomic Characterization of Whole Genome Sequences of Ocular Herpes Simplex Virus Type 1 Isolates Identifies Possible Virulence Determinants in Humans

**DOI:** 10.1167/iovs.64.10.16

**Published:** 2023-07-14

**Authors:** Viet Q. Chau, Aaron W. Kolb, Darlene L. Miller, Nicolas A. Yannuzzi, Curtis R. Brandt

**Affiliations:** 1 Bascom Palmer Eye Institute, University of Miami, Miami, Florida, United States; 2 Department of Ophthalmology and Visual Sciences, School of Medicine and Public Health, University of Wisconsin-Madison, Wisconsin, United States; 3 McPherson Eye Research Institute, University of Wisconsin-Madison, Wisconsin, United States; 4 Department of Medical Microbiology and Immunology, School of Medicine and Public Health, University of Wisconsin-Madison, Wisconsin, United States

**Keywords:** HSV-1, herpes, ocular, keratitis, phylogeny, virulence, drug resistance

## Abstract

**Purpose:**

There are limited data on the prevalence and genetic diversity of herpes simplex virus type 1 (HSV-1) virulence genes in ocular isolates. Here, we sequenced 36 HSV-1 ocular isolates, collected by the Bascom Palmer Eye Institute, a university-based eye hospital, from three different ocular anatomical sites (conjunctiva, cornea, and eyelid) and carried out a genomic and phylogenetic analyses.

**Methods:**

The PacBio Sequel II long read platform was used for genome sequencing. Phylogenetic analysis and genomic analysis were performed to help better understand genetic variability among common virulence genes in ocular herpetic disease.

**Results:**

A phylogenetic network generated using the genome sequences of the 36 Bascom Palmer ocular isolates, plus 174 additional strains showed that ocular isolates do not group together phylogenetically. Analysis of the thymidine kinase and DNA polymerase protein sequences from the Bascom Palmer isolates showed multiple novel single nucleotide polymorphisms, but only one, BP-K14 encoded a known thymidine kinase acyclovir resistance mutation. An analysis of the multiple sequence alignment comprising the 51 total ocular isolates versus 159 nonocular strains detected several possible single nucleotide polymorphisms in HSV-1 genes that were found significantly more often in the ocular isolates. These genes included UL6, gM, VP19c, VHS, gC, VP11/12, and gG.

**Conclusions:**

There does not seem to be a specific genetic feature of viruses causing ocular infection. The identification of novel and common recurrent polymorphisms may help to understand the drivers of herpetic pathogenicity and specific factors that may influence the virulence of ocular disease.

Herpes simplex virus type 1 (HSV-1) is a global pathogen that is estimated to infect more than 3.7 billion people worldwide and can cause a variety of manifestations that confer significant morbidity and mortality.^1^ HSV-1 typically establishes latent infection in the trigeminal ganglia; however, genital HSV-1 infections are increasingly pervasive^2^ and mainly establish latency in the sacral dorsal root ganglia.^3^ Classic HSV-1 viral reactivation can lead to disease anywhere along the trigeminal nerve, including recurrent orolabial vesicles, encephalitis, and keratitis.^4^^,^^5^ Ocular reactivations can be induced by a variety of factors, including fever, ultraviolet light, hormonal changes, stress, and trigeminal nerve manipulation.^6^ HSV keratitis, which has been described for centuries,^7^^–^^11^ is one of the leading causes of poor visual outcomes secondary to infection in developed countries, with more than 230,000 people developing some degree of visual impairment owing to HSV in a single year globally.^12^ An increase in ocular HSV cases has been observed with estimates of more than 500,000 people infected in the United States alone.^7^

Primary herpes simplex ocular infections commonly begin as conjunctivitis, with superficial corneal involvement present in approximately 60% of cases.^6^^,^^13^ However, recurrent ocular infections owing to viral reactivation from latency can cause increasing corneal damage with each reactivation, leading to stromal keratitis, corneal neovascularization, iridocyclitis, and retinitis.^6^^,^^14^ The clouding and damage to the cornea in HSV keratitis is due to the migration of neutrophils, T cells, and macrophages into the site of infection by proinflammatory cytokines and chemokines (including IL-1α, IL-1β, IL-6, IL-8, IFN-γ, TNF-α, macrophage inflammatory protein-2, and macrophage inflammatory protein-1α) secreted by the corneal epithelium and infiltrative immune cells.^14^^–^^18^ After clearance, the infiltrate and clouding are decreased; however, corneal damage remains, subsequently resulting in a permanent decrease in visual acuity.^14^^,^^18^ Ocular morbidity from HSV keratitis is driven partly by antiviral resistance that has led to an increasing recurrence of herpetic ocular infections. After initial episodes of HSV keratitis, approximately 27% recur at 1 year and 50% recur at 5 years, with the risk increasing with the number of prior recurrences.^19^

Three nucleoside analogues (trifluorothymidine, acyclovir, and ganciclovir) are characteristically used for HSV keratitis treatment; however, HSV stromal keratitis is usually treated with an antiviral plus a topical steroid taper for a duration of more than 10 weeks.^20^ Prophylactic treatment with twice daily 400 mg oral acyclovir has been shown to decrease the cumulative probability of recrudescent HSV ocular disease.^21^ Although typically responsive to treatment with acyclovir, up to 6.4% of herpetic keratitis cases in immunocompetent patients are resistant to acyclovir.^22^ Mutations in thymidine kinase (TK) are responsible for up to 95% of acyclovir resistant cases, followed by mutations in DNA polymerase.^23^^–^^25^ More than 130 and 60 polymorphisms in TK and DNA polymerase, respectively, have been confirmed to confer antiviral resistance, with a majority of these being single nucleotide polymorphisms (SNPs).^25^ However, the frequency and implications of these mutations specifically in ocular herpetic disease remains unclear, given limited studies on genetic variation among ocular-derived HSV-1 isolates.

The level of HSV-1 ocular disease pathology in animal models has been shown to be due to three primary factors (host innate immunity, adaptive immunity, in particular CD4^+^ T cells, and the viral strain).^26^^–^^35^ Greater insight into the genetic makeup of individual herpesvirus strains has been aided significantly by next-generation genome sequencing and related multiplexing technology,^36^^–^^42^ resulting in more than 300 HSV-1 genomic sequences being deposited into GenBank. Recently, our group was able to sequence the genomes of multiple HSV-1 recombinants and use a machine learning–based quantitative trait locus technique to map viral genes influencing multiple ocular disease phenotypes in mice.^43^^,^^44^ Although hundreds of HSV-1 sequences are available, only a handful of ocular isolates have been sequenced, making it challenging to perform a powered analysis and draw definitive conclusions on the genetic variability and possible significance of mutations specific to ocular HSV-1 isolates.

To address the limited abundance of available ocular HSV-1 sequences and to understand further the role of genetic mutations in ocular herpetic disease, we sequenced 37 HSV-1 samples from 3 different anatomical ocular origins (conjunctiva, cornea, and eyelid) from patients with a high suspicion of HSV-1 infection. Phylogenetic analysis of the 37 ocular isolates along with 159 nonocular HSV-1 genome sequences demonstrated that the ocular sequences did not cluster together, suggesting that there is no eye-specific HSV-1 strain. Note that one ocular sample, previously identified as HSV-1, turned out to be HSV-2 after sequencing and was excluded from the analysis. Note also that there are more than 300 HSV-1 sequences in GenBank, but many are duplicates or partial genomes and these were excluded, leading to 159 available sequences. Given the prevalence of antiviral therapy in patients with herpes keratitis, we examined the protein sequences of both TK and DNA polymerase from the clinical isolates, revealing several SNPs previously described in the literature as well as multiple novel SNPs. Examination of the full 210-strain (159 HSV-1 nonocular strains plus 51 ocular HSV-1 strains) multiple sequence alignment (MSA) revealed several statistically significant SNPs associated with the ocular derived virus strains. Notably, all the nonsynonymous SNPs mapped to virion-associated components. Collectively, our study offers several human ocular-derived HSV-1 genomic sequences and provides the largest phylogenetic and genomic analysis of ocular-derived HSV-1 isolates in the literature. The significance of the new SNPs is unknown and will require further structural and functional analyses to determine significance for viral pathogenesis and pharmaceutical resistance.

## Methods

### Viruses

Forty-five HSV ocular samples were collected by Bascom Palmer Eye Institute, a university-based eye hospital, from patients thought to be infected with ocular HSV-1 between 2001 and 2015 (Table 1). Of the 45 sequences submitted to genome sequencing, 37 were sequenced successfully, one of which was identified as HSV-2 and was eliminated from further analysis, leaving 36 total HSV-1 sequences (described elsewhere in this article). The approval of the University of Miami Institutional Review Board was obtained before conducting this study, which was performed in accordance with the Health Insurance Portability and Accountability Act of 1996 and adhered to the tenets of the Declaration of Helsinki. Testing logs of the Bascom Palmer Eye Institute Clinical Microbiology Laboratory were reviewed to identify patient samples collected for ocular tissue culture between 2001 and 2015. All ocular samples were obtained in various clinical settings at the Bascom Palmer Eye Institute, including the emergency room, outpatient clinic, and operating room via standard protocol. Samples were taken from three different ocular anatomical sites (conjunctiva, cornea, and eyelid) by ocular surface and eyelid scrapings, followed by recovery in tissue culture. All isolates were deidentified from patient health information. Isolation source information regarding if the infections were primary or recurrent, the severity of the infection, or if antiviral therapy was used is unavailable. Five additional ocular HSV-1 isolates were collected by Dr John Chandler in Seattle, Washington, between 1975 and 1985, plus one oral HSV-1 isolate from Wisconsin (Table 1).

**Table 1.** Table of HSV-1 Isolate Sources, Genome Lengths, and Accession Numbers Sequenced for the Current Study

Species	Strain	Host	Anatomical Isolation Origin	Country of Origin	Isolation Date	Eye	Sex	Method	Genome Length	Accession Number
HSV-1	BP-C1	Human	Conjunctiva	Miami, Florida, USA	2001	OS	M^*^	PacBio Sequel II	152739	OQ724831
HSV-1	BP-C2	Human	Conjunctiva	Miami, Florida, USA	2004	OS	F	PacBio Sequel II	152898	OQ724832
HSV-1	BP-C3	Human	Conjunctiva	Miami, Florida, USA	2004	OD	M	PacBio Sequel II	154130	OQ724833
HSV-1	BP-C4	Human	Conjunctiva	Miami, Florida, USA	2004	OS	M^*^	PacBio Sequel II	152541	OQ724834
HSV-1	BP-C5	Human	Conjunctiva	Miami, Florida, USA	2005	OS	M	PacBio Sequel II	152332	OQ724835
HSV-1	BP-C6	Human	Conjunctiva	Miami, Florida, USA	2005	OS	M	PacBio Sequel II	Sequencing Unsuccessful	
HSV-1	BP-C7	Human	Conjunctiva	Miami, Florida, USA	2005	NA	F	PacBio Sequel II	Sequencing Unsuccessful	
HSV-1	BP-C8	Human	Conjunctiva	Miami, Florida, USA	2005	OS	M	PacBio Sequel II	152674	OQ724836
HSV-1	BP-C9	Human	Conjunctiva	Miami, Florida, USA	2005	OD	M	PacBio Sequel II	152893	OQ724863
HSV-1	BP-C10	Human	Conjunctiva	Miami, Florida, USA	2006	OS	M	PacBio Sequel II	Sequencing Unsuccessful	
HSV-1	BP-C11	Human	Conjunctiva	Miami, Florida, USA	2008	OS	M	PacBio Sequel II	153030	OQ724864
HSV-1	BP-C12	Human	Conjunctiva	Miami, Florida, USA	2010	OD	M	PacBio Sequel II	152858	OQ724865
HSV-1	BP-C13	Human	Conjunctiva	Miami, Florida, USA	2012	OU	M	PacBio Sequel II	Sequencing Unsuccessful	
HSV-1	BP-C14	Human	Conjunctiva	Miami, Florida, USA	2015	OD	M	PacBio Sequel II	152567	OQ724866
HSV-1	BP-C15	Human	Conjunctiva	Miami, Florida, USA	2015	OD	F	PacBio Sequel II	152381	OQ724867
HSV-1	BP-K1	Human	Keratitis	Miami, Florida, USA	2002	OS	M	PacBio Sequel II	153167	OQ724868
HSV-1	BP-K2	Human	Keratitis	Miami, Florida, USA	2009	OS	M	PacBio Sequel II	152311	OQ724888
HSV-1	BP-K3	Human	Keratitis	Miami, Florida, USA	2009	OS	F	PacBio Sequel II	152277	OQ724889
HSV-1	BP-K4	Human	Keratitis	Miami, Florida, USA	2009	OD	F	PacBio Sequel II	151917	OQ724890
HSV-1	BP-K5	Human	Keratitis	Miami, Florida, USA	2010	NA	F	PacBio Sequel II	154135	OQ724891
HSV-1	BP-K6	Human	Keratitis	Miami, Florida, USA	2010	OD	M	PacBio Sequel II	153130	OQ724892
HSV-1	BP-K7	Human	Keratitis	Miami, Florida, USA	2010	OD	M	PacBio Sequel II	Sequencing Unsuccessful	
HSV-1	BP-K8	Human	Keratitis	Miami, Florida, USA	2011	OS	M	PacBio Sequel II	154330	OQ724893
HSV-1	BP-K9	Human	Keratitis	Miami, Florida, USA	2011	OD	M	PacBio Sequel II	153885	OQ724894
HSV-1	BP-K10	Human	Keratitis	Miami, Florida, USA	2011	OD	F	PacBio Sequel II	151502	OQ724895
HSV-1	BP-K11	Human	Keratitis	Miami, Florida, USA	2011	OD	M	PacBio Sequel II	153536	OQ724910
HSV-1	BP-K12	Human	Keratitis	Miami, Florida, USA	2012	OD	F^†^	PacBio Sequel II	152470	OQ724911
HSV-1	BP-K13	Human	Keratitis	Miami, Florida, USA	2014	OD	M	PacBio Sequel II	152231	OQ724912
HSV-1	BP-K14	Human	Keratitis	Miami, Florida, USA	2015	OD	F	PacBio Sequel II	155000	OQ724915
HSV-1	BP-K15	Human	Keratitis	Miami, Florida, USA	2015	OS	F	PacBio Sequel II	173401	OQ724913
HSV-1	BP-K16	Human	Keratitis	Miami, Florida, USA	2015	OD	M	PacBio Sequel II	153180	OQ724914
HSV-1	BP-L1	Human	Eyelid lesion	Miami, Florida, USA	2002	OD	F	PacBio Sequel II	152262	OQ724932
HSV-1	BP-L2	Human	Eyelid lesion	Miami, Florida, USA	2003	OS	M	PacBio Sequel II	153614	OQ724933
HSV-1	BP-L3	Human	Eyelid lesion	Miami, Florida, USA	2003	OS	M	PacBio Sequel II	Sequencing Unsuccessful	
HSV-1	BP-L4	Human	Eyelid lesion	Miami, Florida, USA	2003	OS	M	PacBio Sequel II	151488	OQ724934
HSV-1	BP-L5	Human	Eyelid lesion	Miami, Florida, USA	2003	OD	M	PacBio Sequel II	153093	OQ724935
HSV-2	BP-L6	Human	Eyelid lesion	Miami, Florida, USA	2003	OD	F	PacBio Sequel II	156418	OQ724936
HSV-1	BP-L7	Human	Eyelid lesion	Miami, Florida, USA	2005	OS	F	PacBio Sequel II	Sequencing Unsuccessful	
HSV-1	BP-L8	Human	Eyelid lesion	Miami, Florida, USA	2005	OS	NA	PacBio Sequel II	Sequencing Unsuccessful	
HSV-1	BP-L9	Human	Eyelid lesion	Miami, Florida, USA	2009	OS	F	PacBio Sequel II	153186	OQ724936
HSV-1	BP-L10	Human	Eyelid lesion	Miami, Florida, USA	2009	OS	M	PacBio Sequel II	151308	OQ724937
HSV-1	BP-L11	Human	Eyelid lesion	Miami, Florida, USA	2010	OS	F	PacBio Sequel II	153572	OQ724938
HSV-1	BP-L12	Human	Eyelid lesion	Miami, Florida, USA	2010	OD	M	PacBio Sequel II	153916	OQ724939
HSV-1	BP-L13	Human	Eyelid lesion	Miami, Florida, USA	2011	OD	M	PacBio Sequel II	152962	OQ724940
HSV-1	BP-L14	Human	Eyelid lesion	Miami, Florida, USA	2012	OD	F#	PacBio Sequel II	152371	OQ724941
HSV-1	CJ455	Human	Eye	Seattle, Washington, USA	c. 1980	NA	NA	Illumina HiSeq	152185	OQ724954
HSV-1	CJ462	Human	Eye	Seattle, Washington, USA	c. 1980	NA	NA	Illumina HiSeq	152162	OQ724955
HSV-1	CJ505	Human	Eye	Seattle, Washington, USA	c. 1980	NA	NA	Illumina HiSeq	152204	OQ724962
HSV-1	CJ515	Human	Eye	Seattle, Washington, USA	c. 1980	NA	NA	Illumina HiSeq	152183	OQ724956
HSV-1	CJ858	Human	Eye	Seattle, Washington, USA	c. 1980	NA	NA	Illumina HiSeq	152100	OQ724963
HSV-1	Ikey	Human	Lip	Madison, Wisconsin, USA	2012	NA	M	Illumina HiSeq	152103	OQ724957

F, female; M, male.

* Isolates from same patient.

† Isolates from same patient.

### Cells

Vero cells (CCL-81; ATCC, Manassas, VA, USA) were used for producing viral stocks as well as for generating viral DNA. The cells were propagated in Dulbecco's modified Eagles medium, supplemented with 5% serum (1:1 ratio of bovine calf and fetal bovine serum) plus antibiotics.

### Viral DNA Purification

Viral genomic DNA was isolated from each viral sample, as has been described previously.^45^^,^^46^ Briefly, five confluent TC100 plates of Vero cells were infected with viral stock plus Dulbecco's modified Eagles medium + 2% serum. The infected cells were harvested 24 hours after the monolayer reached 100% cytopathic effect. The cells were centrifuged at 600×*g* for 10 minutes, and the cell pellet was combined with 5 mL of supernatant and subjected to three freeze–thaw cycles. The lysate was then combined with the remaining supernatant and centrifuged at 600×*g* for 10 minutes. The supernatant was then placed on top of a 36% sucrose cushion (in PBS), and centrifuged for 80 minutes at 24,000×*g*. After centrifugation, the supernatant was removed, and the pellet was resuspended in 3 mL of TE (10 mM Tris [pH 7.4], 1 mM EDTA) buffer plus 3 M sodium acetate (0.15 M final concentration [pH 5.5]). The virus prep was then treated with 50 µg/µL of RNAse A and incubated for 30 minutes at 37°C. Proteinase K and SDS (50 µg/µL and 0.1%, respectively) were then added and the preparation was incubated 30 minutes at 37°C. The DNA was then purified by phenol and chloroform extraction. The DNA was then precipitated using ice-cold 95% ethanol and desalted with 70% ethanol, followed by resuspension with sterile water.

### Genomic Sequencing

Before genomic sequencing of the Bascom Palmer HSV viral isolates (Table 1), the quality of the DNA from each sample was measured using a NanoDrop One (ThermoFisher Scientific, Waltham, MA, USA). Next, quantification of the extracted DNA was determined using a Qubit dsDNA High Sensitivity kit (ThermoFisher Scientific). The DNA samples were then diluted and loaded into an Agilent FemtoPulse (Agilent, Santa Clara, CA, USA) electrophoresis system to evaluate DNA size and quality. The samples were converted subsequently into a Pacific Biosciences Microbial Multiplex library according to PN 101-696-100 v07 instructions. Modifications include DNA shearing with Covaris gTUBES (Covaris, Woburn, MA, USA). Library quality was assessed using the Agilent Femto Pulse system, followed by library quantification with the Qubit dsDNA High Sensitivity kit. The library was then sequenced on a PacBio Sequel II (PacBio, Menlo Park, CA, USA), using one SMRT cell and the Sequel Polymerase Binding kit 2.2 at the University of Wisconsin-Madison Biotechnology DNA Sequencing Facility.

After sequencing, the resulting raw PacBio reads were processed and filtered by CCS calling (CSS 6.2.2; https://github.com/PacificBiosciences/ccs), followed by demultiplexing. The demultiplexed reads were then assembled into contigs using hifiasm (https://hifiasm.readthedocs.io/en/latest/index.html#), a de novo PacBio HiFi read assembler.^47^ The viral genomes were then manually assembled from contigs using Mega7.^48^

Six additional HSV-1 isolates, five ocular and one oral isolate (Table 1), were sequenced using the Illumina HiSeq 2000. The sequencing and genome assembly methods have been previously described.^43^^,^^46^

### MSAs, Phylogenetic Network, and Recombination Analysis

Before phylogenetic analysis, the genomic sequences of 168 global HSV-1 isolates were downloaded from the National Center for Biotechnology Information and combined with the 36 sequenced Bascom-Palmer isolates and 6 previously unreleased HSV-1 isolates (Supplementary Table 1). HSV-2 (NC_001798.2) was used as an outgroup. The terminal repeats, TRL and TRS were eliminated from each sequence before alignment. MSA was generated using MAFFT v7.45,^49^ with the FFT-NS-1 option.

For phylogenetic analysis, Splitstree v4.15.1 was used to generate phylogenetic networks^50^ and IQ-TREE v1.6.3^51^ was used to determine the optimal nucleotide substitution models. Genome-based maximum likelihood pairwise distances were calculated using Mega 11.^52^ The pairwise distances were plotted both as a histogram and kernel density plot by using R v4.2.1-arm64^53^ and R Studio v2022.07.2-576^54^ DNAsp v6.12.03^55^ was used to calculate and map SNPs in a given MSA.

Recombination analysis was performed by first generating a genomic MSA of BP-C5, BP-C8, BP-C9, and BP-C14 with MAFFT. The MSA was then subjected to bootscan analysis^56^ with the RDP v4.1 software package,^57^ using a window size of 500 bp and a 250-bp step size.

### Ocular Strain SNPs, Protein Structure Prediction, and Visualization

To determine if any SNPs were associated with the ocular derived HSV-1 isolates, an MSA consisting of the full complement of 210 strains (51 ocular and 159 nonocular) (Supplementary Table 1) versus total ocular strains was generated, without the HSV-2 outgroup. The MSA was scanned and the *P* values of possible ocular associated SNPs were calculated using the Mann–Whitney *U* test (Sigmaplot 11; Systat, San Jose, CA, USA).

Protein structures were either downloaded from Protein Data Bank (https://www.rcsb.org/) or predicted using Alphafold2^58^ in conjunction with Colab (https://colab.research.google.com/github/sokrypton/ColabFold/blob/main/AlphaFold2.ipynb). HSV-1 strain 17 (NC_001806.2) protein sequences were used for the protein structure predictions. The structures were visualized with PyMol (PyMOL Molecular Graphics System, version 1.8.2; Schrödinger, LLC).

## Results

### Sequencing and Genome Assembly

Forty-five ocular isolate samples were taken at the Bascom Palmer Eye Institute from the conjunctiva, cornea, and eyelids from patients believed to be experiencing an ocular HSV-1 infection. DNA samples from all 45 isolates were sequenced using a PacBio Sequel II, resulting in the successful sequencing of 37 strains (Table 1). Of the 45 samples sent for sequencing, 8 were not of sufficient quality and were excluded from the analysis and 1 (BP-L6) turned out to be HSV-2; thus, we used sequences from 36 HSV-1 ocular isolates. The assembled genome sizes varied from 151,308 base pairs (BP-L10) to 173,401 bp (BP-K15) (Table 1). The unusually large size of the BP-K15 genome is due to two large sequences (2819 bp and 8511 bp) inserted into the short repeat genome segment. The 8511-bp sequence seems to be an inverted genome segment corresponding with bases 126,983–132,514 of HSV-1 strain 17 (NC_001806), whereas the approximately 2800-bp insertion is a repeated 5ʹ GCCCTCCCCA 3ʹ sequence. It is unclear if these inserts are due to a sequencing or assembly artifact or represent actual components of the genome. Additional sequencing information detailing average read length and genome coverage is found in Supplementary Table 2.

### Phylogenetic Analysis

**Figure 1.**
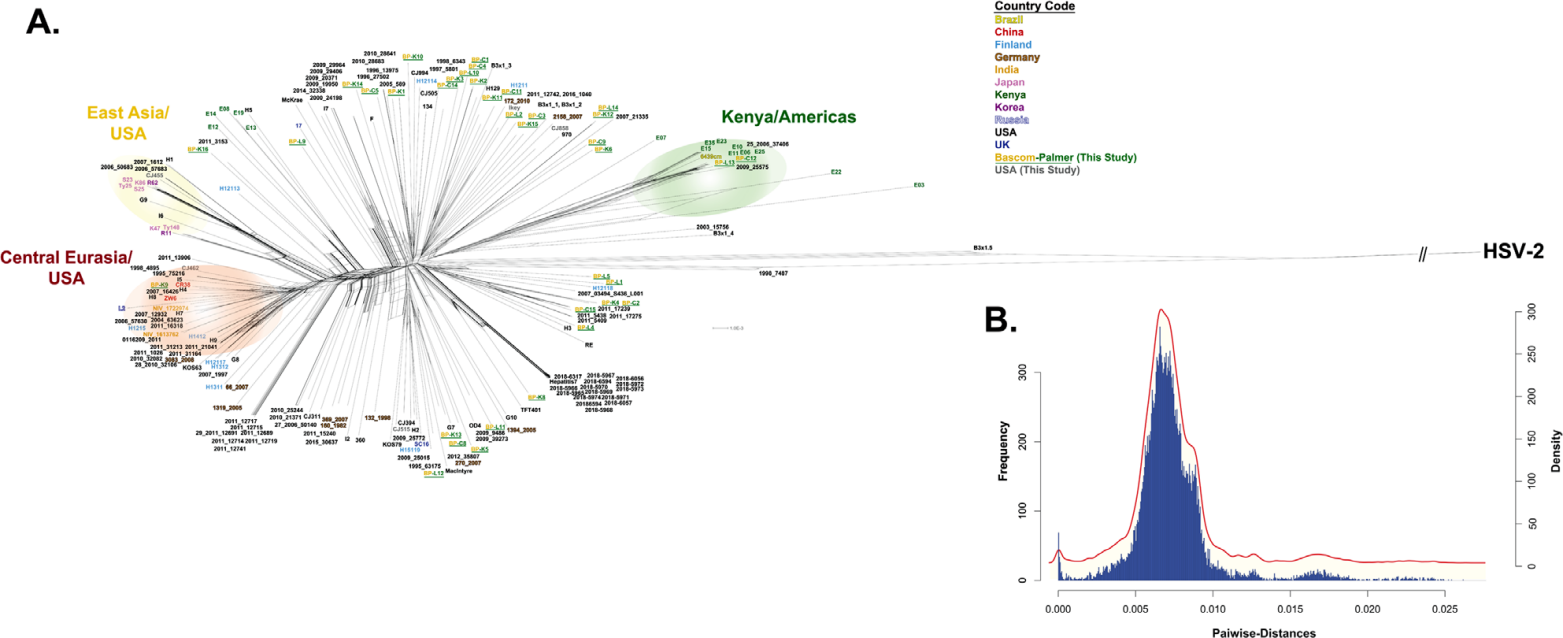
Phylogenetic network and pairwise-distance based kernel density plot of 210 HSV-1 genomes. A phylogenetic network (**A**) was generated with Splitstree, using the GTR+G+I (p-inv = 0.519 and alpha parameter = 0.191, gaps deleted) substitution model from a MSA with 210 HSV-1 genomes plus HSV-2 as an outgroup. The country of origin for each strain is color coded, near the top middle of the figure. The terminal repeats were deleted before sequence alignment. (**B**) Pairwise distance-based histogram and kernel density plot, with frequency corresponding with the histogram and density corresponding with the kernel density plot.

To investigate whether ocular-derived HSV-1 strains group together phylogenetically, an MSA comprising the genomes of the 36 Bascom Palmer HSV-1 ocular isolates, 5 previously unsubmitted ocular strain sequences, 10 ocular strains from GenBank (51 total ocular isolates), and 159 nonocular HSV-1 genomes (GenBank) was generated. A phylogenetic network was constructed (Fig. 1A) because phylogenetic networks can imply recombination and display dissonant phylogenetic signals. The network in Figure 1A shows that the 51 ocular isolates are distributed mostly randomly and do not form an ocular clade. In general, Figure 1A also shows that most of the HSV-1 strains form a long branching star-like pattern with little overall structure.

Pairwise distances were calculated next, then plotted (Fig. 1B) to determine if an objective, genomic, distance-based clade cutoff could be calculated for the network. No clade cutoff could be established, owing to only one main pairwise distance peak (Fig. 1B). To calculate a distance cutoff, two peaks must be present, with the midpoint between the peaks establishing the cutoff. Despite the lack of a cutoff and general lack of overall phylogenetic structure, three clades or groupings could be distinguished: Kenya/Americas, East Asia/USA and Central Eurasia/(Fig. 1A). Two Bascom Palmer samples, BP-C12 and BP-13, grouped into the Kenya/Americas clade, close to a Brazilian isolate (6439 cm). Furthermore, BP-C12 and BP-13, along with several Kenyan strains exhibit very short branch lengths. The Central Eurasian/USA clade comprises sequences ranging from China in the east, through India, and Germany in the West, with relatively moderate branch lengths compared with the rest of the network. Only one Bascom Palmer ocular strain sorted in the Central Eurasian/USA clade, and none in the East Asian/USA clade.

**Figure 2.**
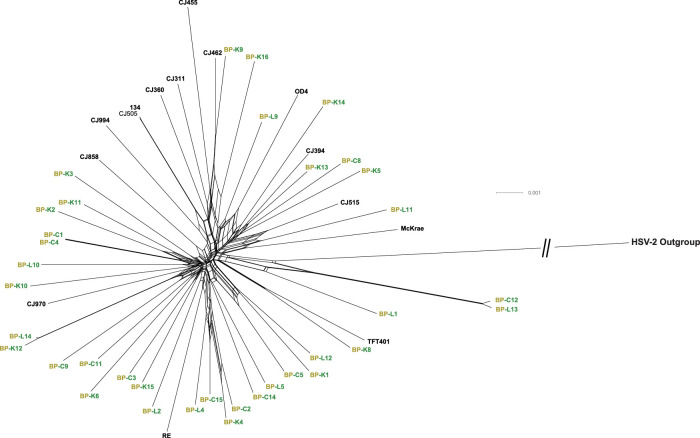
Genome based phylogenetic network of the 51 total available ocular isolates. The phylogenetic network was constructed with Splitstree using the GTR+G+I (p-inv = 0.683 and alpha parameter = 0.349, gaps deleted) substitution model and HSV-2 as an outgroup. The Bascom Palmer–derived ocular isolates are colored orange and green.

Next, a genome-based phylogenetic network exclusively consisting of 51 total ocular isolates was produced to examine if the Bascom Palmer strains clustered according to anatomical area of isolation (conjunctiva, cornea, and eyelid) (Fig. 2). The results showed that the Bascom Palmer isolates did not cluster according to anatomical source; additionally, the phylogenetic network was essentially devoid of a clade-like structure (Fig. 2). An examination of both phylogenetic networks indicated three closely related pairs of Bascom-Palmer isolates: BP-C1/BP-C4, BP-L14/BP-K12, and BP-C12/BP-L13. According to the source data (Table 1), the BP-C1/BP-C4 pair were isolated from the conjunctiva of the same patient in different years (2001 and 2004, respectively). A separate patient was the source of the BP-L14/BP-K12 pair, albeit isolated the same year, however, from the eyelid and cornea. A search for DNA polymorphisms in both viral strain pairs with DNAsp showed that aside from a handful of insertions and deletions (INDELs) in the large repeat regions, no SNP differences were detected in the pairs (data not shown). The BP-C12/BP-L13 strain pair were isolated from different patients in 2010 and 2011, respectively. An analysis of the pair with DNPsp found approximately 130 nucleotide polymorphism differences (data not shown), which is far below the previously reported 600 to 700 nucleotide SNPs between HSV-1 strains.^36^

### Recombination Analysis

**Figure 3.**
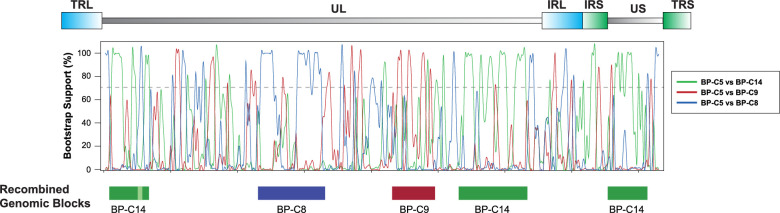
Representative example of recombination in the Bascom Palmer ocular isolate dataset. A map of the HSV-1 genome is located at the top. The bootscan function of RDP4 was used to generate the graph where BP-C5 was scanned against BP-C8, BP-C9, and BP-C14. Recombined genomic blocks are displayed at the bottom. A window size of 500-bp and 250-bp step sizes was used for the analysis.

Recombination in HSV-1 is highly pervasive^46^^,^^59^^–^^61^; as such, we sought to confirm the presence of recombination in the Bascom Palmer ocular isolate dataset. To detect representative recombination, the genome of isolate BP-C5 was bootscanned against those of BP-C14, BP-C9, and BP-C8. Bootscan detected three genomic blocks of BP-C14, and one segment each of BP-C8 and C9 contributing to the BP-C5 genome (Fig. 3), implying that recombination has occurred.

### TK and UL30 Polymerase SNP Analysis

**Figure 4.**
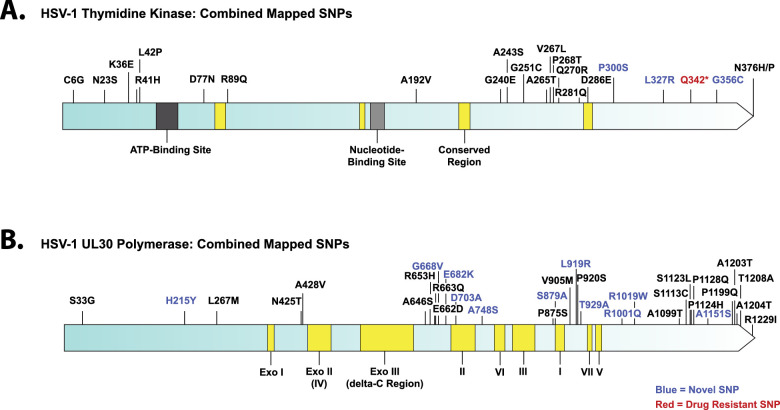
Mapped TK and UL30 polymerase SNPs from the Bascom Palmer ocular dataset. TK is shown in (**A**), and UL30 polymerase is below (**B**). Previously described SNPs are shown in black, novel SNPs are in blue, and known drug-resistant SNPs are in red.

HSV keratitis is usually treated with antivirals, including acyclovir, sometimes for long periods, resulting in complications involving possible antiviral resistance. Acyclovir resistance is overwhelmingly caused by SNP changes in TK, although the UL30 polymerase can be involved. We looked for evidence of possible antiviral resistance in the 36 HSV-1 Bascom Palmer samples by logging nonsynonymous SNPs located in TK and UL30 (Fig. 4). In the Bascom Palmer dataset, we found 23 nonsynonymous SNPs in TK and 32 nonsynonymous SNPs in UL30. Four of the SNPs detected in TK were novel, and 11 of the UL30 SNPS have not been described. One SNP in TK from the BP-K14 isolate encoded a Q342stop, which has been shown previously to result in acyclovir resistance.^25^ A log of the TK and UL30 SNPs detected in each individual strain can be found in Supplementary Table 3. None of the other TK or UL30 SNPs found in the dataset were known to be associated with drug resistance when compared with the current literature.^24^^,^^25^^,^^62^^,^^63^ To better visualize how the novel SNPs in TK and UL30 may affect structure or function, the novel SNPs were mapped both to previously determined crystal structures as well as alphafold2 protein prediction. Alphafold2 prediction was used because some of the SNPs are located in uncrystallized areas. The TK and UL30 polymerase structures with mapped novel SNPs are found in Supplementary Figures S1 and S2, respectively. None of the TK novel SNPs map close to the active site and are located mainly to exterior points (Supplementary Fig. S1). Although none of the novel SNPs in UL30 map close to the active site, several (G668V, E682K, and R1019W) map to alphafold2 predicted random coil “stanchions” (Supplementary Fig. S2), which may surround the DNA strand, and plausibly interact with it.

### SNPs and INDELs Leading to Gene Truncations

We next wanted to explore the dataset for SNPs or INDELs that would lead to full or partial gene deletions or additions. The survey of the sequences revealed that both the BP-K4 and BP-K14 isolates carried a 5ʹ deletion of the ICP47 (UL12) immediate early gene, leading to a complete deletion (Table 2). A SNP in the UL3 gene of the BP-L5 isolate resulted in a near complete deletion of the gene, encoding only seven amino acids before a premature stop. Other genes containing significant deletions compared with wild type were UL4 (BP-K10), VP13/14 (BP-C12), US2 (BP-C5), and UL36 (BP-L13). Other SNPs and INDELs lead to smaller gene truncations in γ134.5 (BP-C8), UL5 (BP-C14), UL32 (BP-L11), UL36 (BP-C15), and UL37 (BP-L2).

**Table 2.** SNPs and INDELs in Bascom Palmer Isolates Leading to Either Full or Partial Gene Deletions

Isolate	Location	Change	Gene	Protein	WT Protein Length	Isolate Protein Length
BP-C5	134726	T to - (fs)	US2	US2	291	146
BP-C8	1109	C to A (stop codon)	RL1	Y134.5	241	239
BP-C12	102367	- to A (fs)	UL47	VP13/14	693	269
BP-C14	12668	- to G (fs)	UL5	UL5	882	881
BP-C15	72137	- to G (fs)	UL36	VP1/2	3139	3024
BP-K4	145515	Deletion of 5’ end	US12	ICP47	88	0
BP-K10	12308	68 bp deletion	UL4	UL4	199	79
BP-K14	47157	G to A (stop codon)	UL23	TK	376	341
BP-K14	147569	Deletion of 5’ end	US12	ICP47	88	0
BP-L2	82127	GC to – (fs)	UL37	UL37	1123	1119
BP-L5	11104	C to A (stop codon)	UL3	UL3	224	7
BP-L11	68481	GGGT to —- (fs)	UL32	UL32	596	542
BP-L13	74108	C to - (fs)	UL36	VP1/2	3139	2149

### Detection of Possible Ocular Strain Related SNPs

**Figure 5.**
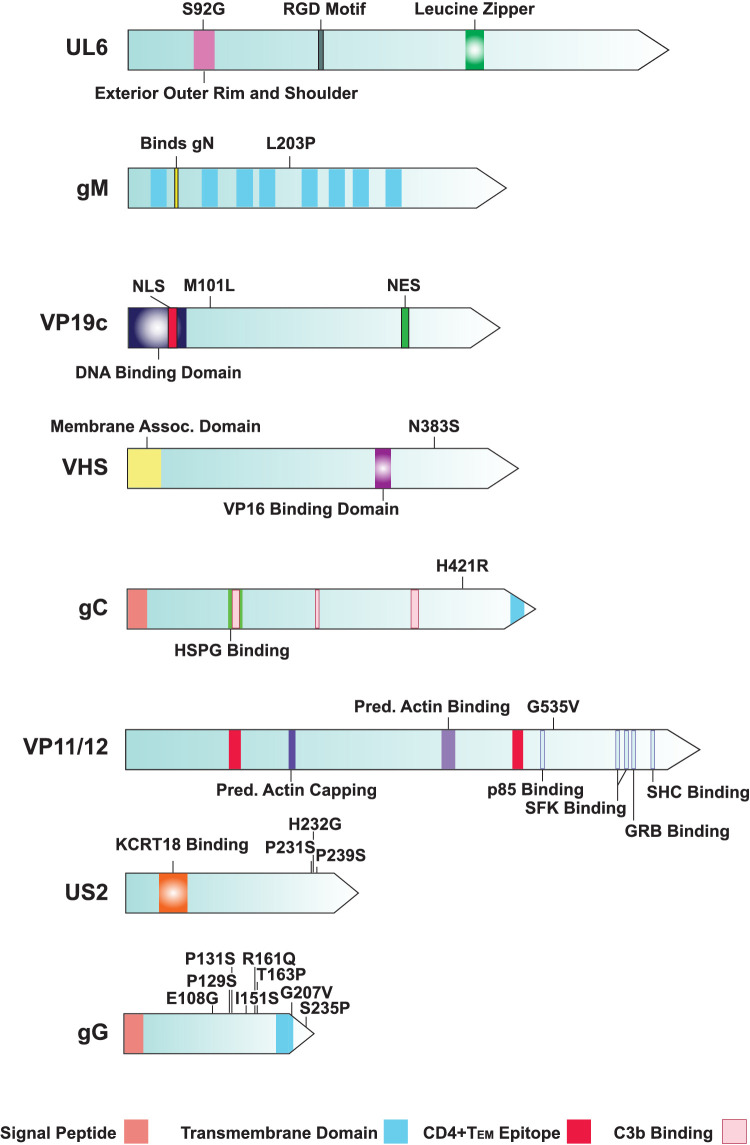
Protein maps of significant nonsynonymous SNPs identified in ocular HSV-1 strains. The individual SNPs are mapped on each protein with the functional regions or motifs shown either within the protein or in the key below.

Although the genome-based phylogenetic analysis of 51 ocular strains plus 159 nonocular strains (Supplementary Table 1) failed to recover an ocular strain-based clade (Fig. 1A), this does not necessarily preclude the possibility that individual SNPs may be biased in ocular strains. A MSA comprising 210 strains (51 ocular and 159 nonocular) was scanned for possible SNPs that may be biased in the ocular strains, with *P* values calculated for any detected SNPs. The analysis revealed 15 significant SNPs in the dataset that were present significantly more often in the ocular isolates (Table 3). Five of these SNPs encoded synonymous changes in the UL5, UL6, UL8, gH, and ICP8 genes, and two additional SNPs were located in noncoding areas; the US1 mRNA and the intergenic region between US9 and US10. Statistically significant nonsynonymous SNPs mapped to the UL6, gM, VP19c, VHS, gC, VP11/12, US2, and gG genes (Table 3). We next mapped the nonsynonymous SNPs in each gene to each respective protein to determine if the SNPs mapped to function regions or motifs (Fig. 5). Figure 5 shows that none of the SNPs map to known major functional or protein–protein interaction areas.

**Table 3.** SNPs Identified as Being Significant in Relation to Ocular Versus Nonocular Isolates

MSA Coordinates	Strain 17 Coordinates	SNP	Ocular/Nonocular, n SNP; % Incidence	Gene	Synonymous/Nonsynonymous	Mann-Whitney *P* Value
8418	14650	C/T	Ocular: 41/10; 80%	UL5	Synonymous	<0.001
			Nonocular: 52/104; 33%			
11502	15175	T/G	Ocular: 33/18; 65%	UL6	Synonymous	<0.001
			Nonocular: 55/101; 35%			
11731	15404	T/G	Ocular: 34/18; 65%	UL6	Non; UL6 S92G	<0.001
			Nonocular: 64/92; 41%			
15459	18859	C/A	Ocular: 28/23; 55%	UL8	Synonymous	0.002
			Nonocular: 119/37; 76%			
21685	23504	A/G	Ocular: 33/18; 65%	gM/UL10	Non; gM L203P	<0.001
			Nonocular: 44/112; 28%			
47520	44734	T/G	Ocular: 35/16; 68%	gH/UL22	Synonymous	<0.001
			Nonocular: 68/88; 43%			
66940	59331	C/T	Ocular: 33/18; 64%	ICP8/UL29	Synonymous	<0.001
			Nonocular: 60/96; 38%			
98245	84831	A/C	Ocular: 32/19; 63%	VP19c/UL38	Non; VP19a M101L	0.004
			Nonocular: 60/96; 38%			
106552	91486	T/C	Ocular: 36/15; 71%	VHS/UL41	Non; VHS N384S	<0.001
			Nonocular: 57/99; 35%			
118750	97573	G/A	Ocular: 35/16; 69%	gC/UL44	Non; gC H421R	<0.001
			Nonocular: 62/94; 40%			
121203	99353	C/A	Ocular: 37/14; 72%	VP11/12 UL46	Non; VP11/12 G535V	<0.001
			Nonocular: 57/99; 35%			
220997	133939	A/C	Ocular: 31/20; 61%	US1	Noncoding	<0.001
			Nonocular: 47/109; 30%	mRNA		
221344^*^	134216^*^	G/A^*^	Ocular: 42/9; 82%	US2	Non; US2 P231S, H232G, P239S	<0.001
			Nonocular: 79/77; 51%			
224835^*^	137013^*^	C/T^*^	Ocular: 40/11; 78%	gG/US4	Non; gG E111G, P132S, D134S, I153S,	0.002
			Nonocular: 73/71; 52%		R164Q, T166P, G210V, S238P	
235529	143989	TT/AA	Ocular: 38/13; 74%	I.G US9/US10	Noncoding	<0.001
			Nonocular: 49/107; 31%			

* A group of multiple SNPs.

To examine if greater insight could be gained by studying the SNPs in relation to protein structure, alphafold2 was used to predict the protein structures of gM, VP19c, VHS, gC, VP11/12, Us2, and gG (the crystal structure of UL6 has been determined). Next, the detected SNPs were mapped to the known or predicted protein structures (Supplementary Fig. S3). The mapped SNPs in UL6, VHS, and US2 map to the exterior or the protein; moreover, the G535V SNP in VP11/12 maps to a random coil back face containing several SRC family kinase binding sites. The mapped M101L SNP of VP19c is positioned close to a predicted N-terminal random coil containing both the nuclear localization sequence as well DNA-binding properties. Additionally, the glycoprotein M and G predicted structures both have SNPs mapped to the cytoplasmic side of their transmembrane domains. Glycoprotein G contains a series of linked SNPs, seven of which map to the outside of the membrane (Supplementary Fig. S3). Further, the ectodomain of gG is predicted to be almost entirely random coil, which is in stark contrast with the predicted structure of gC. The gC H421R SNP maps to a potential hinge point, and maps away from the predicted head containing both the heparan sulfate proteoglycan and C3b binding areas.

## Discussion

Although next-generation genome sequencing has facilitated the sequencing of hundreds of HSV-1 genomes deposited into GenBank, only 10 ocular-derived sequences were available at the onset of this project. This paucity of data constrains investigating viral genetic factors that may influence the phylogenetics and virulence of ocular HSV-1 strains. In the current study, we isolated and sequenced 36 additional HSV-1 ocular strains from patients at the Bascom Palmer Eye Institute in Miami, Florida, with an additional 5 unreleased genomes from ocular derived strains. These ocular HSV-1 isolate genomes were analyzed along with 159 nonocular sequences. The findings indicate that (i) ocular isolates did not form a clade, (ii) the ocular isolates did not cluster according to anatomical origin (conjunctiva, cornea, or eyelid), (iii) only one SNP detected in the Bascom Palmer dataset encoded a known acyclovir resistance mutation, and (iv) statistically significant nonsynonymous SNPs found more often in ocular strains mapped to the UL6, gM, VP19c, VHS, gC, VP11/12, US2, and gG genes.

### Phylogenetic Analysis

Although multiple studies into HSV-1 phylogenetics have been conducted,^64^^–^^67^ none have focused on ocular versus nonocular isolates owing to data scarcity. To the best of our knowledge, this phylogenetic analysis, which includes 51 total ocular strains, comprises the greatest number of total HSV-1 strains yet carried out (210 strains). First, the results show that, on a genomic level, the ocular-derived HSV-1 strains do not form a phylogenetic clade (Fig. 1), nor do the Bascom Palmer isolates cluster according to the site of anatomical origin (Fig. 2). This result is unsurprising, given that similar results have been reported with fellow alphaherpesviruses EHV-1^68^ and EHV-4,^69^ where viral isolates did not cluster according to disease phenotype. The incongruence between phylogeny and disease phenotype at the genomic level is likely because a variety of factors, including host genetics and innate immunity, contribute to a disease phenotype,^26^^,^^70^^–^^73^ versus phylogeny, which is primarily based on genetic distance.

The 210 HSV-1 strain phylogenetic network incorporates a cosmopolitan assortment of global isolates, including strains isolated from Latin Americans (Fig. 1; Supplementary Table 1). The majority of the viral strains in the phylogenetic network exhibit long branch lengths, branching out from roughly the center, resembling a porcupine or star, with generally little clade structure. This finding contrasts with previous studies with considerably smaller datasets,^41^^,^^64^^,^^65^^,^^67^ but is similar to what has been observed previously in HSV-2.^74^ The absence of phylogenetic structure has been observed in several organisms^75^^–^^78^ and is associated with population mixing and expansion. This finding is consistent with the global human population dynamics of the last several hundred years, especially in Eurasia and the Americas, thereby influencing HSV-1 phylogenic signals. Despite the overall lack of phylogenetic structure, three clades or groups (Kenya/Americas, East Asia/USA, and Eurasia/USA), could still be discerned. An unanswered question is why a majority of the network is unstructured, yet three organized groups can still be detected. This factor is especially apparent for the Central Eurasia/USA group, which contains strains essentially spanning the entire length of the Eurasian continent. The long, branching, star-like pattern observed in Figure 1A strongly implies extensive recombination and population expansion, as well as a complex evolutionary history. This observation is important for future phylogenetic studies with HSV-1, because significant caution should be taken when attempting time-measured phylogenetic analysis, because time-measured analysis is often complicated by recombination and population expansion.^79^

Two of the Bascom Palmer isolates, BP-C12 and BP-L13, which were isolated from separate patients (Table 1), seem to be closely related (Fig. 2), with only approximately 130 nucleotide SNPs separating them, as opposed to the typical (approximately 700 SNPs).^36^ A close examination of the placement of these strains in a global context place them within a group of Kenyan strains exhibiting very short branch lengths (Fig. 1A). The Bascom Palmer Eye Institute serves patients across the globe, and we are unsure of the personal origins of the patient sources for BP-C12 and BP-L13. We are uncertain how to best account for the short branch lengths and low genetic diversity of the viruses in this viral subgroup. Additional sequencing and patient data may help to elucidate this factor in the future.

Two additional pairs of Bascom Palmer isolates were collected from the same individual: BP-C1/BP-C4 and BP-K12/BP-L14 (Table 1). The BP-C1/BP-C4 isolates were both isolated from the cornea of the same patient, but in different years. The BP-K12/BP-L14 pair were likely isolated at the same time (2012), albeit from different anatomical sites. For both pairs, aside from minor INDEL differences (data not shown), the sequences were essentially identical. This result contrasts somewhat with a recent study by Rathbun et al.,^80^ which found high HSV-1 genome sequence conservation among genital infection transmission partners. This study also detected within-host sequence diversity in 8 of 10 participants over time; however, the amount of diversity differed between individuals. Additional work is required to determine if the low sequence diversity found in the related ocular sample pairs described here is due to low sample number or a property of ocular infections.

Phylogenetic work over the past several years has noted the curious phylogenetic position of the widely used laboratory HSV-1 strain KOS.^42^^,^^64^ These previous phylogenetic studies with smaller datasets found that the widely used KOS63 strain clusters with Asian viruses.^42^^,^^64^ In the context of the much larger dataset presented here, KOS63 was placed into the Central Eurasian/USA clade; moreover, the closest sequences to KOS63 were those from Germany, Finland, and the United States (Fig. 1A). Thus, the previous assignment of KOS63 seems to be incorrect, pointing out caveats or issues related to the use of limited numbers of strain sequences for phylogenetic analysis.

### Virulence Gene Analysis

Although the ocular derived HSV-1 ocular strains did not cosegregate at the genomic level, which was not unexpected, individual SNPs are more likely to influence virulence. This outcome is backed by studies in gallid herpesvirus 2,^81^ Marek's disease virus,^82^ EHV-1,^83^^,^^84^ and HSV-1,^43^^,^^44^ which have identified individual SNPs influencing viral virulence. In the Bascom Palmer ocular HSV-1 dataset, several SNPs and INDELs were found which led to premature stops in various genes (Table 2). Western blots of UL32 and ICP47 were conducted to confirm the sequencing data; however, the results were inconclusive (data not shown). Specifically, the anti-ICP47 antibody detected multiple nonspecific proteins and was deemed invalid. Although the UL32 antibody (curtesy of Dr Sandra Weller, University of Connecticut) was effective, owing to an apparent molecular weight of 68 kda and the UL32 putative mutant only lacking approximately 50 amino acids, size differences were not apparent. Assuming the accuracy of sequencing is correct, it is difficult to predict the effect of the deletion in essential gene UL32 in cell culture or patients. The functional domains of UL32 have not been mapped in detail; however, alphfold2 structure prediction comparison (Supplementary Fig. S4) shows that deletion of the 54 C-terminal amino acids does not affect the central core of the protein; however, four outer alpha helices are missing. It is possible that the basic function of UL32 is still preserved. The two frameshifts leading to deletions in UL36 are likely either sequencing errors, or due to complementation by another strain. Schipke et al.^85^ previously showed that viruses containing similar UL36 C-terminal deletions resulted in inability of the mutant viruses to form plaques. The remaining gene deletions are in nonessential genes, and it is possible that some of the isolates containing the deletions may have come from immunocompromised patients, resulting in a lower bar for the virus to cause pathology, however that clinical data is unavailable. Another potential explanation is complementation by viruses with wild-type alleles in the patient sources.

A scan of the MSA consisting of 51 ocular strains and 159 nonocular strains identified SNPs in several genes that were found significantly more often in the ocular isolates. Synonymous SNPs were found in the UL5, UL6, UL8, gH, and ICP8 genes. Additionally, the UL5 (helicase subunit) synonymous SNP presented the greatest difference between ocular and nonocular strains (80% of ocular strains vs 33% of nonocular strains) (Table 3) in the dataset. Although synonymous SNPs were once thought of having mostly silent effects,^86^ recent work has shown that synonymous changes often have a strong phenotypic influence.^87^^,^^88^ Having stated this, at this time, we are unable to speculate if and how the identified synonymous SNPs may impact their respective genes or HSV-1 ocular disease.

**Figure 6.**
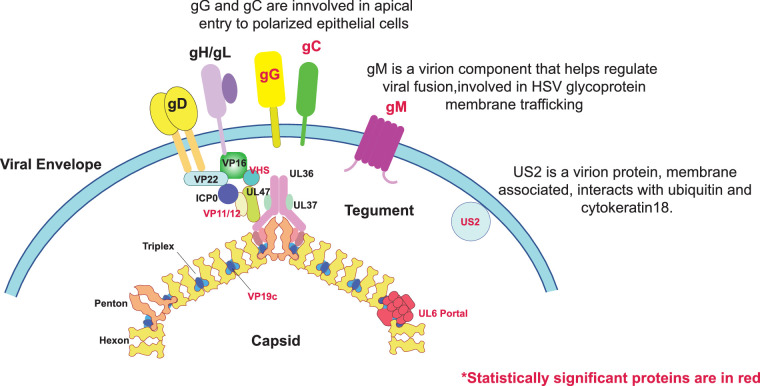
Summary figure of the proteins containing nonsynonymous significant SNPs associated with ocular HSV-1 strains. Each of the significant proteins is written in red.

Significant nonsynonymous SNPs were detected in eight genes (UL6, gM, VP19c, VHS, gC, VP11/12, US2, and gG) (Table 3). These results are preliminary, and care needs to be taken not to overinterpret them, with downstream work required, which is beyond the scope of the current study. Partially validating this analysis, the VHS, gC, VP11/12 and gG have been identified previously as ocular virulence determinants.^43^^,^^44^^,^^89^^–^^92^ It is also noteworthy that all of the eight identified genes encode virion proteins, which is summarized in Figure 6. Despite the preliminary nature of the SNP analysis, some inferences as to how some of the identified SNPs may influence ocular infection can be made. Glycoproteins G and C have been shown to affect entry at the epithelial apical surface,^90^^,^^93^ which could impact corneal surface viral entry. Related to this, the gM glycoprotein (Table 3; Fig. 6), which with gN regulates the viral fusion,^94^ contains SNPs on the cytoplasmic side of the membrane (Supplementary Fig. S3), an area that is linked to both protein maturation and trafficking.^95^^–^^97^ A series of linked SNPs were found in gG, most of which mapped to the extracellular portion (Fig. 5; Supplementary Fig. S4). The chemokine binding activity of gG is complex,^98^^,^^99^ inducing multiple changes including CXCR4 nanoclustering. It is possible the multiple detected SNPs could influence gG's chemokine binding affinity in the corneal epithelium. Glycoprotein G is not the only significant gene in the dataset affecting innate immunity, because US2 (Table 3, Fig. 6), a membrane associated ubiquitin binding protein,^100^ also regulates nuclear factor-κB signaling.^101^ Another identified protein, VP11/12, that, like most viral proteins, is multifunctional, is required for AKT activation, and interacts with STING and TANK binding kinase 1.^102^^,^^103^ The identified SNP (G535V) in VP11/12 physically maps to the predicted random coil back C-terminus of the protein (Supplementary Fig. S3), which contains several SRC kinase binding sites and would be consistent with G525V possibly influencing protein–protein interactions. The proteins identified in this analysis may provide specific targets to treat ocular HSV-1 infections in the future.

## Supplementary Material

Supplement 1

Supplement 2

Supplement 3

Supplement 4
