# Supplementary material for: Phylogenetic and Genomic Characterization of Whole Genome Sequences of Ocular Herpes Simplex Virus Type 1 Isolates Identifies Possible Virulence Determinants in Humans
Source: Invest Ophthalmol Vis Sci. 2023 Jul 14;64(10):16. doi: 10.1167/iovs.64.10.16 (PMC10353747; doi:10.1167/iovs.64.10.16)
Supplement: Supplement 1 [file iovs-64-10-16_s001.pdf]

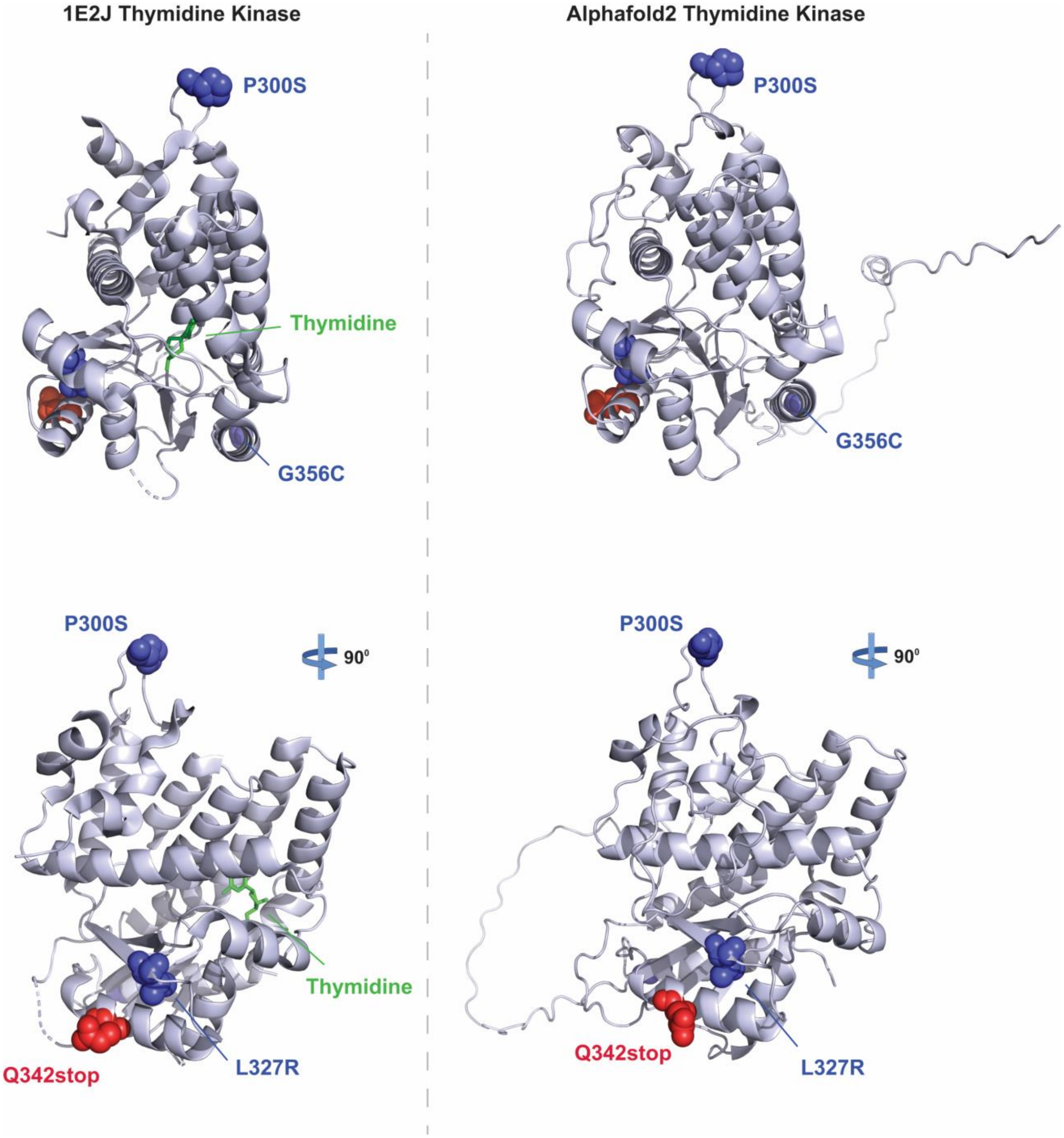

Supplementary Figure 1. The crystalized (1E2J) and predicted (Alphafold2) protein structure of thymidine kinase. Novel SNPs are mapped in blue and the drug resistant Q342stop SNP is red.

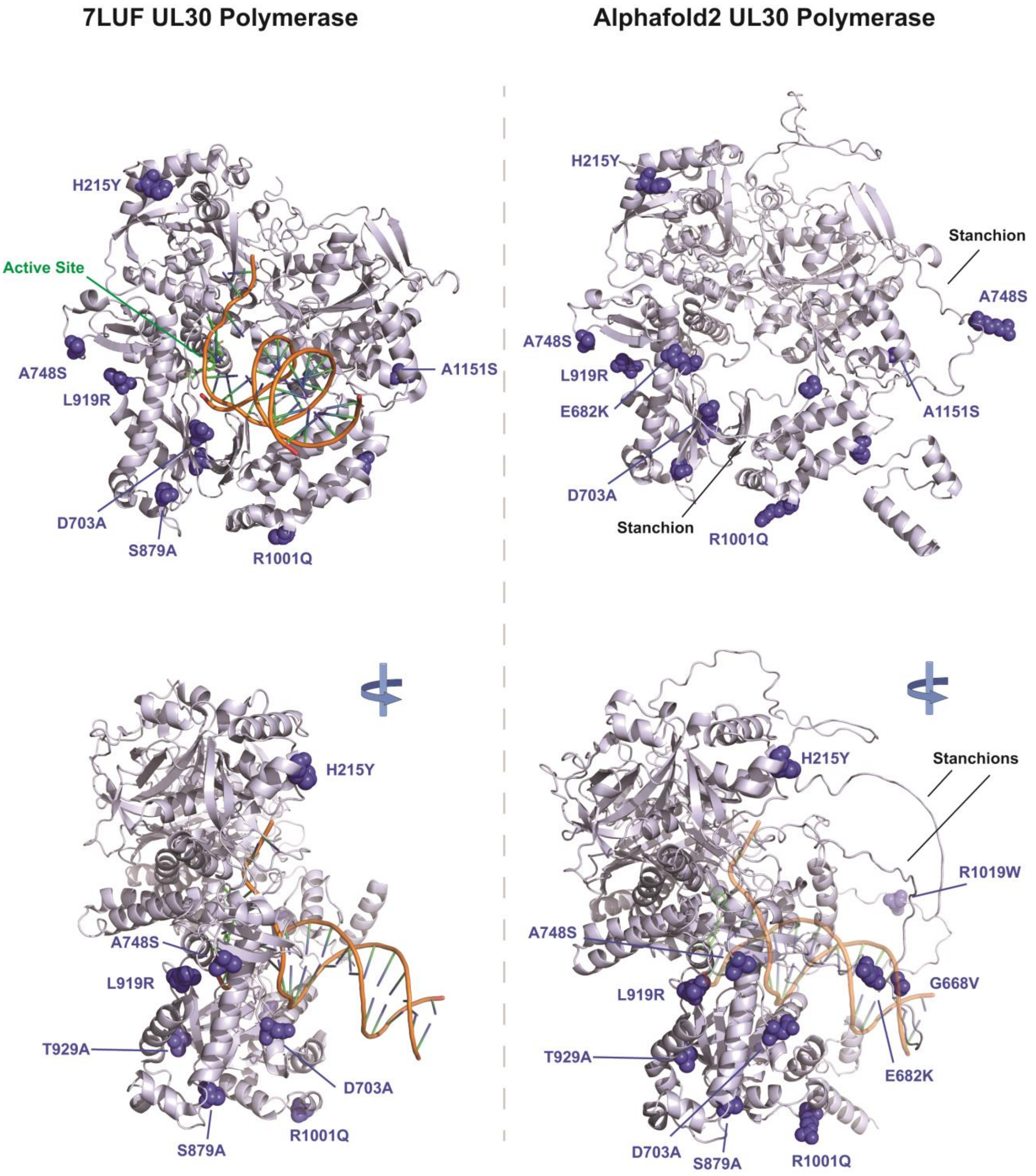

Supplementary Figure 2. The crystalized (7LUF) and predicted (Alphafold2) protein structure of HSV-1 UL30 polymerase. Novel SNPs are mapped in blue. The DNA backbone is orange, and superimposed onto the predicted structure.

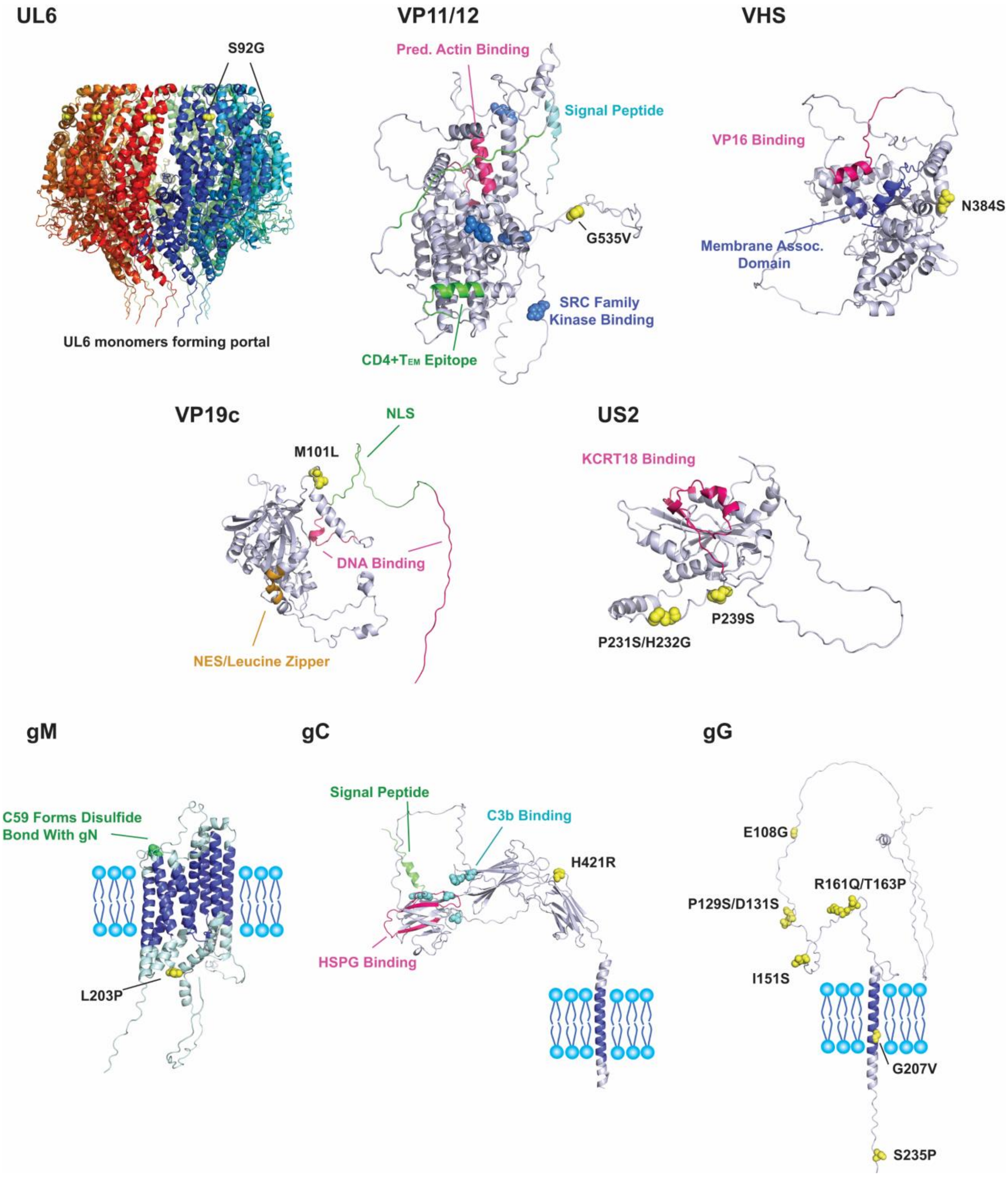

Supplementary Figure 3. The determined (UL6) and predicted (remaining) protein structures of proteins containing significant ocular HSV-1 associated SNPs. SNPs in each protein are

denoted by black writing and yellow amino acid spheres. Transmembrane motifs are blue, and the cell membrane is represented by light blue balls and tails.

Strain 17 UL32 Alphafold2 Structure

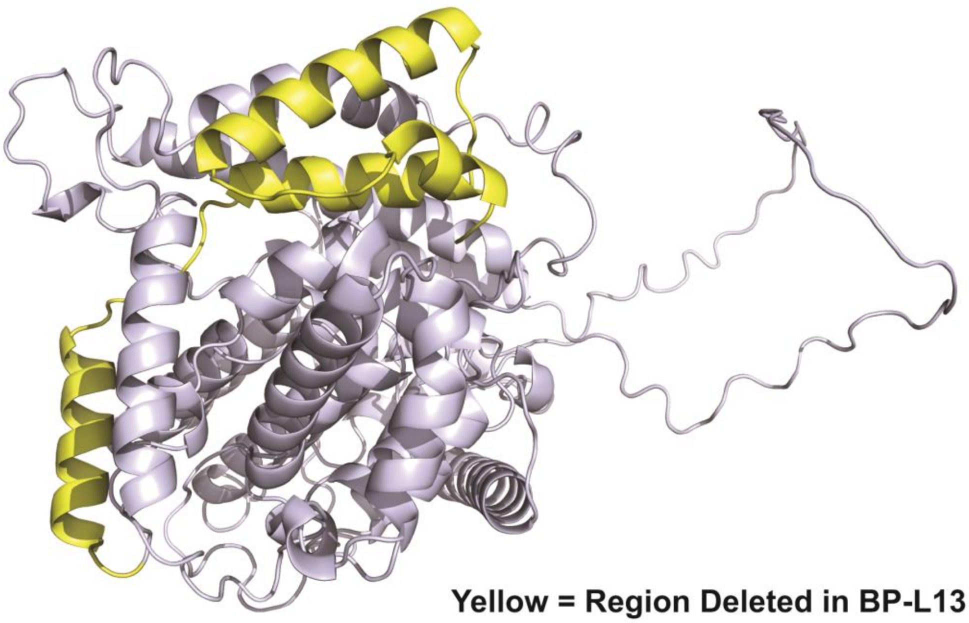

BP-L13 UL32 Alphafold2 Structure

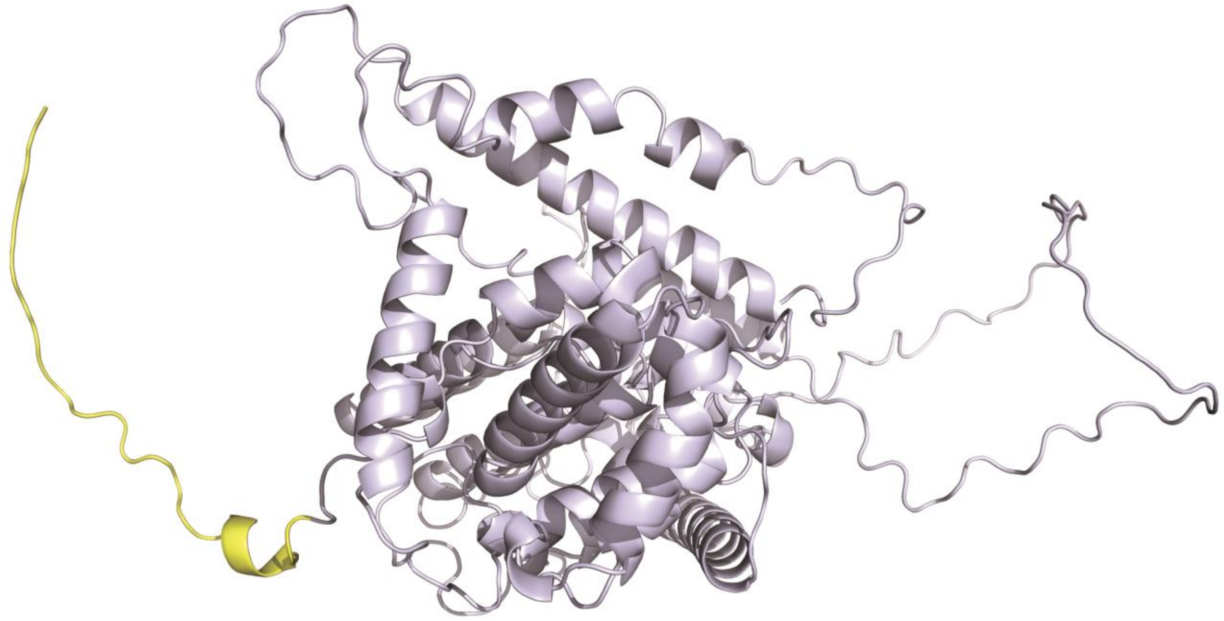

Yellow = Amino Acids Encoded Following Frame Shift

Supplementary Figure 4. The predicted Alphafold2 structures of Strain 17 and BP-L13 UL32. The wild-type Strain 17 structure is at the top and the areas in yellow are putative deletions in BP-L13. The lower model is of BP-L13 UL32 and the yellow region contains amino acids encoded subsequent to a putative frameshift.
